# Histological and histochemical features of the mature female reproductive tract of local breed dog *(Canis familiaris)*

**DOI:** 10.5455/javar.2024.k835

**Published:** 2024-12-27

**Authors:** Dhyaa Ab Abood, Mohammed Sulaiman Dawood, Noor Hussein Yousif, Abdulkarim Jafar Karim

**Affiliations:** 1 Department of Anatomy & Histology, College of Veterinary Medicine, University of Baghdad, Baghdad, Iraq; 2 Iraq Natural History Research Center and Museum, University of Baghdad, Baghdad, Iraq; 3 Tropical Biological Research Unit, College of Science, University of Baghdad, Baghdad, Iraq

**Keywords:** Bitch, cervix, endometrium, histochemical uterine gland

## Abstract

**Objective:**

Many studies focused on clinical cases such as ovariohysterectomy of bitches and scarcely mentioned the histological features. The present study describes the cytoarchitecture characteristics of a local dog’s mature adult reproductive tract.

**Materials and Methods:**

Sixteen samples of uterus and cervix were obtained from local breed bitches to conduct this study. The organs were processed according to routine histopathological protocol and stained with hematoxylin and eosin, Masson’s trichrome, and combined Alcian blue (2.5 pH) and PAS (AB-PAS) stains.

**Results:**

The mature endometrium formed numerous short epithelial folds and epithelial crypts composed of mucous cells and cuboidal cells. The core of the endometrium is composed of fibrous tissue containing fibroblasts with discernible active uterine glands. The myometrium is constructed by thick, circularly, and longitudinally oriented layers of smooth muscle fibers. The AB-PAS stain of the uterine glands revealed light-neutral glycoprotein. The cervix had a very thick wall and displayed numerous huge mucosal folds, covered by ciliated and non-ciliated pseudo-stratified epithelium. The tissue core of the cervix was very thick, and composed of highly cellular, highly vascular, and non-glandular fibrous connective tissue. Layers forming the muscularis of the cervix are composed of circularly, obliquely, and longitudinally oriented smooth muscle bundles. The cervical epithelial cells revealed a light film of neutral glycoprotein that covered the epithelial surface, and the goblet cells denoted strong acidic mucopolysaccharide.

**Conclusions:**

The current study concluded that the most mature nonpregnant local breed dogs during the proestrus and estrus phases had an active endometrial architecture that is suitable for the management of reproduction.

## Introduction

The local breed dog, as a carnivore species, was ignored in veterinary histology; most studies focused on surgery and other branches of veterinary science. The uterus in a bitch is composed of two horns and a well-demarcated body and is classified as a bicornuate [1]. The bitch is a non-seasonal, mono-estrus animal with spontaneous ovulation only twice a year and with an atypical, postovulatory oocyte maturation [2]. Many studies focused on a clinical case such as ovario-hysterectomy of bitches depending on the causes [3]. Bitch has a unique estrous cycle that is considerably longer than other domestic animals, and they are considered non-seasonal anestrus of variable follows each estrous cycle [4]. The epithelium of the uterus is represented by normal lipid-laden endometrial cells during the met-estrus, and di-estrus phases and plays a role in pregnancy or pseudo-pregnancy cases [5].

During pro-estrus, the uterine glands in the beagle dog breed show marked proliferation, and the endometrial stroma is edematous. The myometrium is thick and associated with hypertrophy. During estrus, increased stromal collagen results in the thickened eosinophilic endometrial stroma with myometrial characteristics similar to those of the pro-estrus phase. At di-estrus, the uterus has a thick myometrium with hypertrophied smooth muscle cells and a thick endometrium. The columnar lining cells are eosinophilic with a fine vacuolated appearance. At an-estrus, the uterus is atrophic and has basophilic endometrial stroma, with compact myometrium [6]. During the estrus cycle of a bitch, the tumor necrosis factor was found in urine epithelium and stromal fibroblast normally found during the oestrus cycle and also with changes associated with the early steps of blastocyst invasion [7]. Van Cruchten et al. [8,9] reported that a majority of the bitches showed a cycle of di-estrus/an-estrus phase with slight variation in the duration of an-estrus in dog breeds [10]. In canines, false placental hyperplasia is a nonmicrobial disease with peculiar features. The presence of a layer of connective tissue mast cells in addition to superimposed inflammatory cellular exudate refers to cystic endometrial hyperplasia or pyometra [11]. Reproductive histology is important for successful reproductive management and for the application of assisted reproductive biotechnology, so this study has been done to characterize the chemo-cytoarchitecture of the reproductive organs of female dogs.

## Materials and Methods

### Ethical approval

All study manners are planning-approved and certified according to (Approval No. PG/2300) by the Animal Care and Use Committee on December 11, 2023, at the College of Veterinary Medicine, University of Baghdad, Iraq.

### Animal tissue samples

Uteri and cervi from 16 adult non-pregnant bitches were collected from hopeless surgical cases brought to private veterinary clinics in the Baghdad province. This study was performed in the Department of Anatomy, Faculty of Veterinary Medicine, University of Baghdad, between March and September 2022. Vaginal swabs were collected from bitches to detect the phase of estrus. Tissue specimens were taken from the cervix, body, and uterine horns; the samples were washed down with 0.9% normal saline, and fixation was done by using 10% formalin buffered saline for 2 days and processed for routine histopathology [12,13]. Paraffin blocks were sectioned at 4 μm using an automatic rotary microtome, tissue sections stained with hematoxylin and eosin (H&E) and Masson’s trichrome (MT), combined Alcian blue (2.5 pH) and PAS (AB-PAS) stains, examined, and the histological images were captured by SC 35 camera (Olympus^®^). The tunica of the uterus and cervix were subjected to the histometrical measurement, scored, and analyzed according to Suad et al. [14] using the Fiji image analyzer system^®^.

## Results

**Figure 1.**
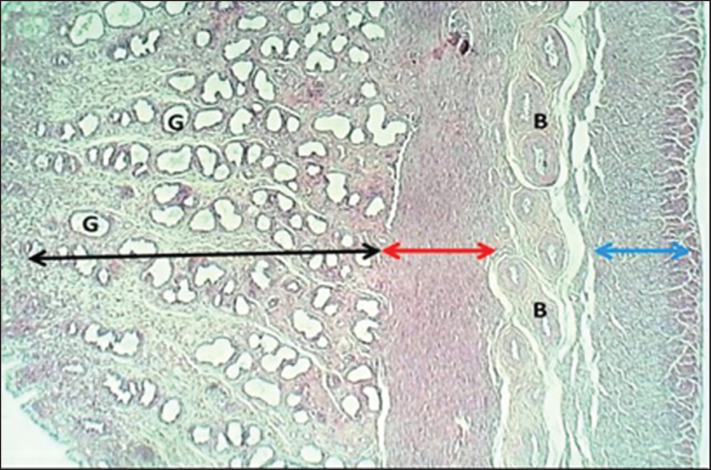
A cross-section of the uterus (mature bitch) shows (black double head arrow) endometrium, (G) uterine glands, (red double head arrow) inner circular layer of myometrium, (blue double head arrow) outer longitudinal layer of myometrium, with blood vessels (B). H&E stain.

**Figure 2.**
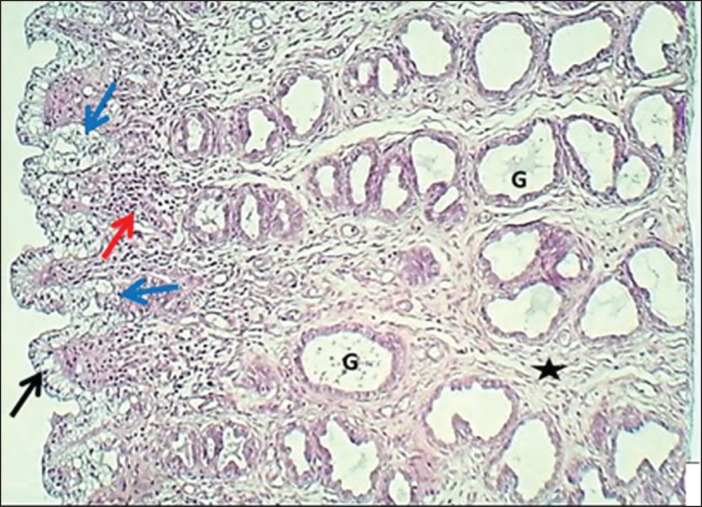
Section of endometri um (mature bitch) shows (black arrow) short epithelial folds, (blue arrows) epithelial crypts, (red arrow) cellular lamina propria, (G) hypertrophied uterine glands & fibrous connective tissue (asterisk). H&E stain.

**Figure 3.**
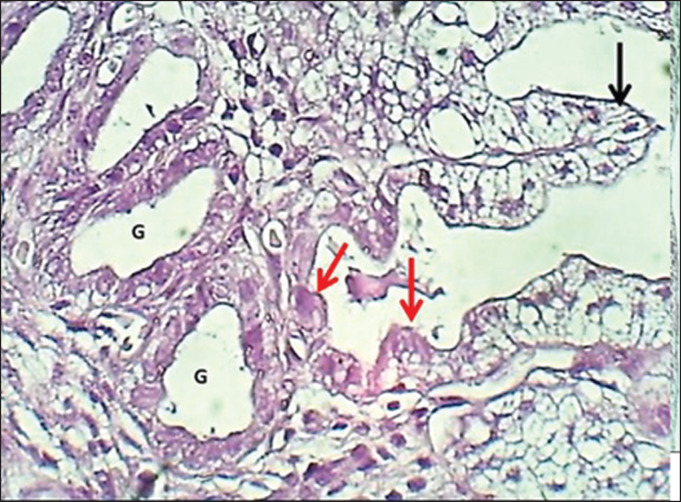
Section of the endometrium (mature bitch) shows (black arrow) mucous secreting epithelial cells, (red arrow) zymogen secreting cells & (G) uterine glands within fibrous connective tissue. H&E stain.

**Figure 4.**
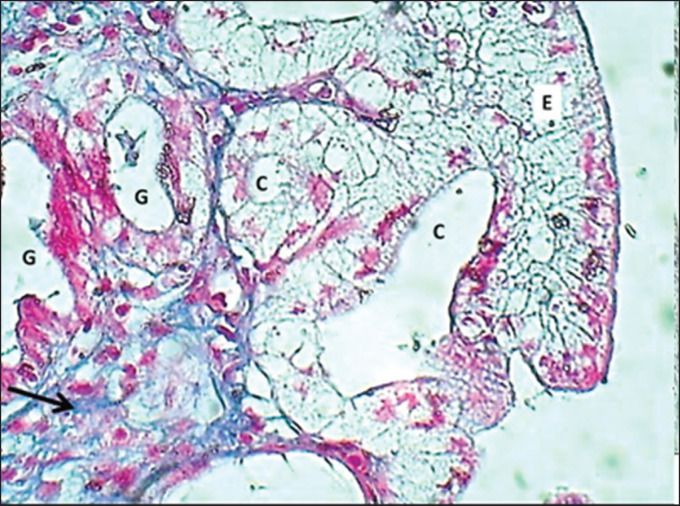
Section of the endometrium (mature bitch) shows; epithelium (E), epithelial crypts (C), uterine glands (G), stromal collagen fibers (arrow) & arteriole (black arrow). MT stain.

**Figure 5.**
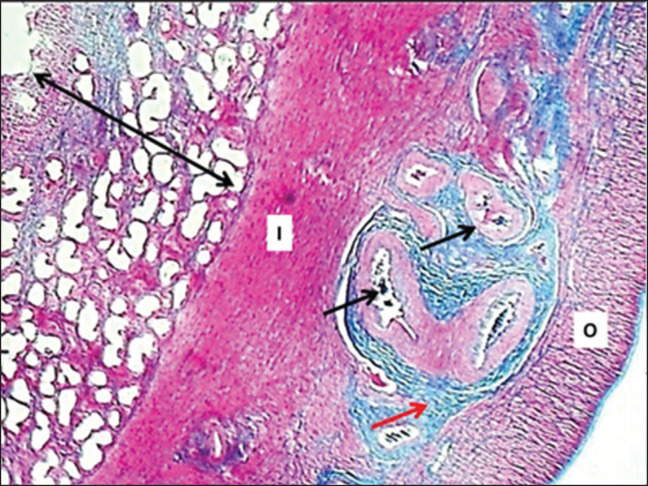
Section of uterine wall (mature bitch) shows: endometrium (double arrowhead), inner circular layer of myometrium (I), outer longitudinal layer of the myometrium (O), fibrous connective tissue with numerous arteries (arrows). MT stain.

**Figure 6.**
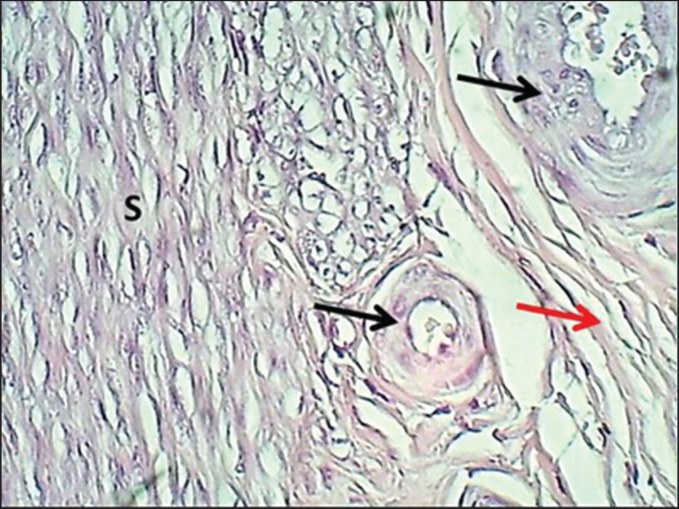
Section of myometrium (mature bitch) shows: (S) smooth muscle fibers of the inner circular layer of the myometrium, (black arrows) arteries, (red arrow) collagen fibers. H&E stain.

**Figure 7.**
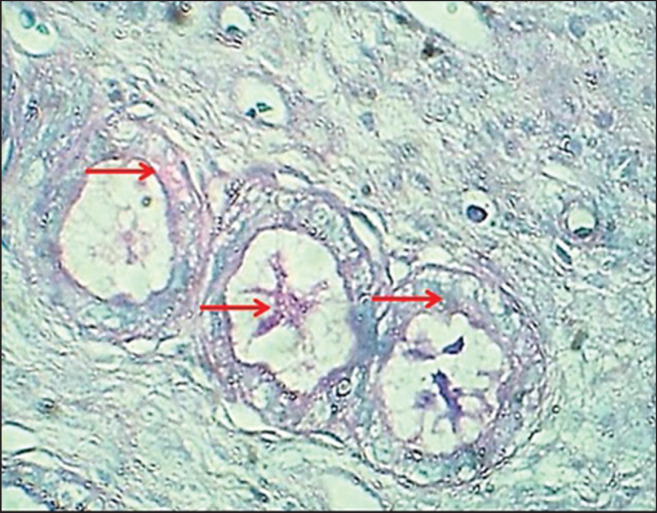
Section of uterine glands (mature bitch) shows (red arrows) neutral glycoprotein secretion. AB-PAS stain.

The study revealed that 10 (62.5%) and 6 (37.5%) of bitches were during the proestrus and estrus phases, respectively. The results revealed that the uterus of local breed bitches had a chunky wall and had three major tunicae: endometrium, myometrium, and premetrium, as shown in Figure 1. The first layer, the endometrium, is characterized by a well-demarcated epithelial mucosa forming numerous short folds that extend into the lamina propria to form epithelial crypts (Figs. 2 and 3). The epithelial mucosa consisted of simple columnar epithelium that had two types of cells: mucus-secreting columnar cells, which were the predominant type, had lightly stained cytoplasm with H&E stains containing mucous secretion. The next type of epithelial cells was a few darkly stained cuboidal cells that lined the epithelial crypts and had eosinophilic cytoplasm (Fig. 3). Endometrial lamina propria was made up of highly cellular fibrous connective tissue occupied by numerous active hypertrophied uterine glands that had a prismatic arrangement. The uterine glands were simple tubular glands that were built up by eosinophilic simple cuboidal epithelial cells (Figs. 3 and 4). The myometrium is composed of double chunky laminae of the non-striated (smooth) muscle fibers, inner circularly and outer longitudinally ordinated bundles, nourished with a highly vascularized tissue composed of irregular bundles of collagen fibers and small arteries (Figs. 5 and 6). The histometrical results revealed that the mean thickness of endometrium was (599.48 ± 5.59 µm), the mean epithelial height was (18.25 ± 1.82 µm), the mean diameter of epithelial crypts was (46.69 ± 2.75 µm), the mean diameter of the uterine gland was (40.224 ± 1.79 µm), and the mean thickness of myometrium was (432.322 ± 6.39 um). The uterine glands showed a light neutral glycoprotein when stained by an AB-PAS stain (Fig. 7).

**Figure 8.**
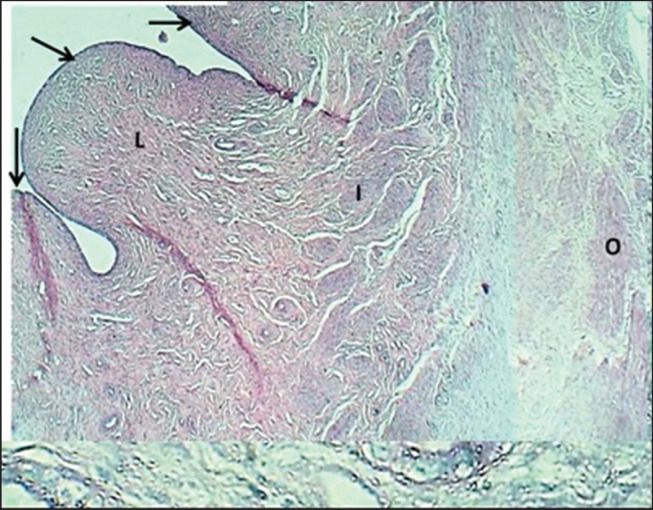
Section of the cervix (mature bitch) shows: (arrows) mucosal folds, (L) non-glandular fibrous lamina propria, (I) inner muscularis & outer muscularis layer (O). H&E stain.

**Figure 9.**
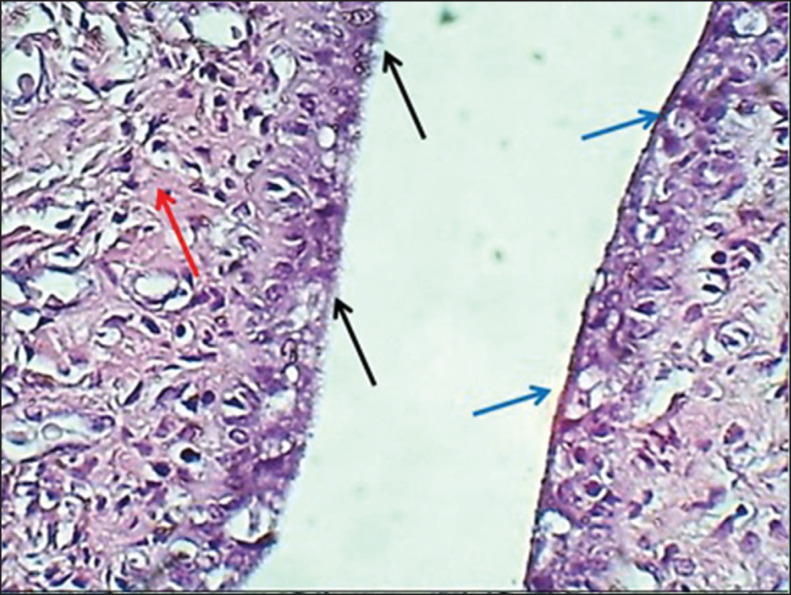
Section of the mucosa of the cervix (mature bitch) shows: (black arrows) ciliated pseudostratified epithelium, (blue arrows) non–ciliated epithelium & collagen bundles of lamina propria with blood vessels (red arrows). H&E stain.

**Figure 10.**
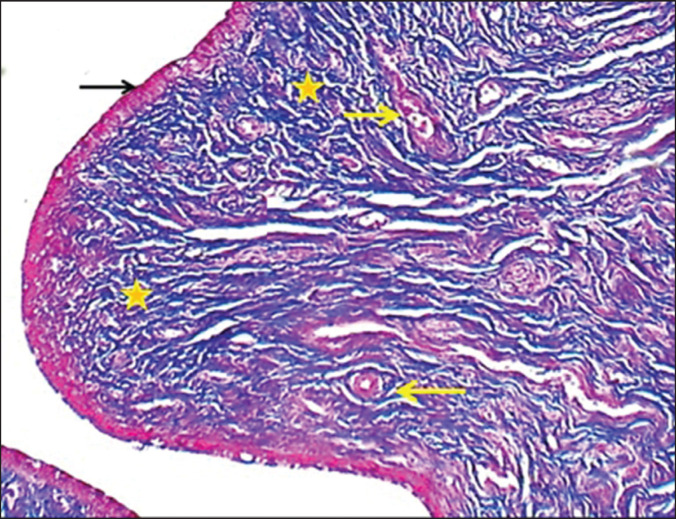
Section of cervix folds (mature bitch) shows: (black arrow) epitheli um, (asterisks) collagen bundles of non-glandular lamina propria & blood vessels (yellow arrows). MT stain.

**Figure 11.**
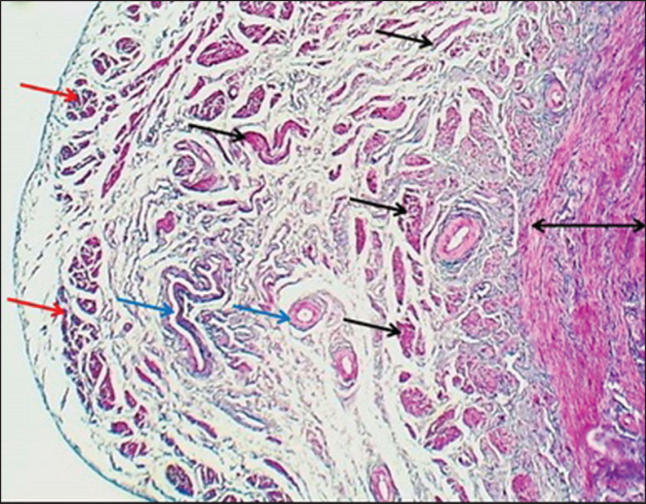
Cross section of cervix wall (mature bitch) shows (double heads arrow) inner layer, (black arrows) middle oblique layer, (red arrows) outer longitudinal smooth muscle layer of tunica muscularis & loose connective tissue with blood vessels of venous plexus (blue arrows). MT stain.

**Figure 12.**
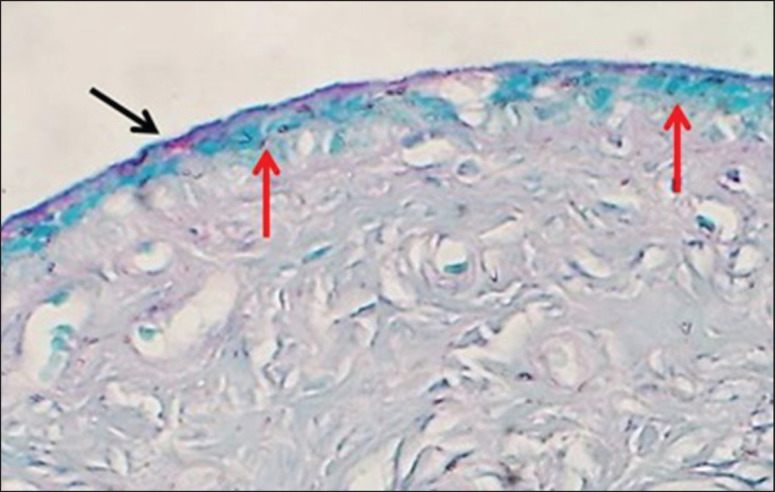
Section of cervix (mature bitch) shows thin neutral glycoprotein secretion (black arrows) & goblet cells containing acidic mucopolysaccharide (red arrows) AB-PAS stain.

The cervix of local breed bitches revealed an extremely chunky wall and had three major tunica: mucosa, muscularis, and serosa (Fig. 8). The tunica mucosa of the cervix displayed numerous huge pyramidal mucosal folds (Fig. 8) that were covered by pseudostratified epithelium that consisted of non-secretion-producing epithelial cells (ciliated) and secretion-producing epithelial cells (non-ciliated). The ciliated epithelial cells showed eosinophilic cytoplasm while the non-ciliated epithelial cells (mucus-secreting cells) showed light-stained cytoplasm (Fig. 9). Mucosal folds of lamina propria consisted of extremely chunky cellular fibrous connective tissue and numerous fibroblasts, fibrocytes, and blood vessels; glandular tissue was not seen. The venous plexus had a marked pattern in the deepest parts of the tissue core (Figs. 10 and 11). The muscularis externa of the cervix was a trilaminar smooth muscle structure comprised of circularly, obliquely, and longitudinally oriented bundles that were separated from each other and supported by loose connective tissue with blood vessels (Fig. 11). The histometrical results of the cervix revealed that the means of the height and thickness of the mucosal folds were (454.88 ± 4.40 µm and 12.021 ± 2.17 µm), respectively, and the mean epithelial height was 17.66 ± 1.37 µm and the mean thickness of the tunica muscularis was 389.52 ± 3.06 µm. By using an AB-PAS stain, the cervix epithelial cells showed a light film of neutral glycoprotein that covered the epithelial surface, and the goblet cells denoted strong acidic mucopolysaccharide (Fig. 12).

## Discussion

The uterus of local breed bitches showed a very chunky wall consisting of triple tunicae, which were similar to that seen in goats [15], rats [16], and rabbits [17], who mentioned that the uterus has three tunicae; endometrium (mucosa), myometrium (muscularis) and perimetrium (serosa). The current study confirmed that the features of pro-estrus and estrus endometrium of local breed bitches were displaying numerous epithelial folds. This result coincided with the result of Rathod and Dixit [15] in goats and Akinloye and Oke [16] in rats, who mentioned that the epithelium formed low longitudinal folds. On the contrary, the current result disagrees with the observation of Abd-Elkareem [17] in rabbits, Singh et al. [18] in goats, and Santos et al. [19] in guinea pigs. The current result suggests that the pro-estrus phase was related to the active physiological status of the endometrium related to the level of estrogen that causes an increase in the secretory activities of the epithelium.

In canines, the endometrium is not replaced during each cycle as it occurs in humans when the endometrium is divided into an uppermost stratum functional, which is sloughed off at menstruation and renewed during each menstrual cycle [20]. Our study revealed that the endometrium at the pro-estrus phase was covered by simple columnar epithelium which predominantly secretory cells. This result is similar to that recorded in women by Dutta and Talukdar [21], who mentioned that the epithelium consisted of secretory non-ciliated columnar cells during the postmenopausal phase. A similar finding was recorded by Abd-Elkareem [17] in the uterus of rabbits and Santos et al. [19] in guinea pigs. Simmons et al. [20] declared that the glandular epithelium is a cuboidal type. Akinloye and Oke [16] mentioned that the epithelium of the endometrium was between pseudo-stratified and simple columnar epithelium. Moreover, Rathod and Dixit [15] mentioned that the epithelial mucosa ranges from simple columnar epithelium in goats to pseudo-stratified columnar epithelium in some places.

The histometrical measurements of the present study confirmed that the mean thickness of the endometrium during the pro-estrus phase coincided with Dutta and Talukdar [21], who mentioned that the endometrium thickness increased at estrus while thinning and atrophied in postmenopausal age. The endometrial lamina propria appeared as a very thick layer consisting of highly cellular fibrous connective tissue occupied by numerous prismatically arranged active uterine glands. This result agreed with the results of Akinloye and Oke [16] in African giant rats during the oestrus cycle, Abd-Elkareem [17] in rabbits, and Santos et al. [19] in guinea pigs. Rathod and Dixit [15] mentioned that the uterine lamina propria in goats has two zones: the superficial layer, which has vascular loose connective tissue, and the deep layer, which has less cellular loose connective tissue. The current result suggests that the cellular component of lamina propria was stromal active fibroblasts, which are related to the proliferation of supporting stromal cells in addition to important growth factors that are secreted during the proestrus phase. As well as that recorded by Dutta and Talukdar [21], the uterine glands were simple tubular glands that built upward vertically to the luminal surface. Our result showed that the myometrium had chunky internal circular layers and chunky external longitudinal layers of the smooth muscle fibers held by separated, well-vascular fibrous connective tissue. This result was in agreement with that recorded by Rathod and Dixit [15], Akinloye and Oke [16], Abd-Elkareem [17], and Santos et al. [19].

The cytoarchitecture of the cervix in bitches was different in comparison to other animals. The present results revealed numerous huge, simple, pyramidal mucosal folds that are similar to those recorded by Goericke-Pesch et al. [22] in canines, Huchzermeyer et al. [23], and Vink and Myers [24] in mares. On the contrary, Juli et al. [25] mentioned that the cervix of Gayo mare displayed branched cervical folds categorized into primary, secondary, and tertiary folds. Rezaian & Hamedi [26] showed that loose connective tissue of the uterus of Caspian mares possesses eosinophils, neutrophils, fibroblasts, mast cells, fibrocytes, and lymphocytes, as well as simple tubular glands that were noticed at the bottom of subaltern folds and filled the lamina propria in the area of the endo and mid cervix and missed in the exo-cervix.

Our result confirmed that the epithelium of the cervix lined by pseudo-stratified epithelium had two types of cells, ciliated and non-ciliated cells. Such a result was similar to that recorded by Goericke-Pesch et al. [22] in canines. Doğan et al. [27] described that the cervix of the goat was covered by pseudo-stratified columnar epithelium. Dissimilarly, Juli et al. [25] recorded that the cervix of a mare is composed of columnar cells that are arranged into ciliated and secretory cells. This result disagreed with Huchzermeyer et al. [23], who mentioned that the lining epithelium has three types of cells. Also, Dutta and Talukdar [21] recorded that the epithelium of the cervix was simple columnar epithelium to non-keratinized stratified squamous epithelium. The free epithelial cell surface shows a clear, obvious border of kinocilia along the total cervical canal. Similarly, the cervix in humans, primates, and ruminants is padded by one layer of tall columnar cells [28]. The present finding revealed that the average measurement of the cervical folds was higher than that recorded in the cervix of mares (17.5 ± 1.7 μm) by Huchzermeyer et al. [23].

The current result showed that the lamina propria is a fibrous, glandular connective tissue with fibroblasts and fibrocytes. The cervix displayed ideal epithelial crypts, so the lamina propria revealed well-developed cervical glands [29] in the description of the structure of the sow cervix. In ruminants, the cervix has well-developed mucous simple tubular glands [30]. On the other hand, Huchzermeyer et al. [23] observed a well-vascularized plexus of veins and venules supporting the occlusive function of the cervical canal in the form of a cavernous body. Moreover, cervical mucosa in monkeys, marmosets, and possums have large amounts of clefts and tubular glands [28]. The bitch cervix revealed the absence of cervical glands, which are dissimilarly formed by the lining epithelium in gilt [28]. Our result revealed a well-developed venous plexus deeper to lamina propria coinciding with that mentioned in mares [26,27].

Similarly, a dense fibrous connective tissue of collagen fibers builds up in the cervical wall in small ruminants [25]. The solidity of the cervical canal is due to collagen fiber, whereas elastic fibers are plentiful in the cervical mucosa, submucosa, and muscular layer in small ruminants [30]. Our finding revealed that the tunica muscularis of the cervix is composed of inner, middle, and outer layers of smooth muscle arranged in circular, oblique, and longitudinal patterns, respectively, supported by vascularized loose connective tissue. This result disagrees with that of Doğan et al. [27] in goats, who mentioned only inner circular and outer longitudinal layers.

## Conclusion

The current study concluded that the most mature nonpregnant local breed dogs were during the proestrus and estrus phases, which had active endometrial cytoarchitecture that was suitable for the management of reproduction.

## List of abbreviations

AB-PAS, Alcian blue (2.5 pH) and Periodic Acid Schiff; H&E, Hematoxylin and Eosin; MT, Masson’s trichrome.
